# Emergence of a Stable Cortical Map for Neuroprosthetic Control

**DOI:** 10.1371/journal.pbio.1000153

**Published:** 2009-07-21

**Authors:** Karunesh Ganguly, Jose M. Carmena

**Affiliations:** 1 Department of Electrical Engineering and Computer Sciences, University of California Berkeley, Berkeley, California, United States of America; 2 Helen Wills Neuroscience Institute, University of California Berkeley, Berkeley, California, United States of America; 3 Department of Neurology, University of California San Francisco, San Francisco, California, United States of America; 4 Program in Cognitive Science, University of California Berkeley, Berkeley, California, United States of America; University of Minnesota, United States of America

## Abstract

Cortical control of neuroprosthetic devices is known to require neuronal adaptations. It remains unclear whether a stable cortical representation for prosthetic function can be stored and recalled in a manner that mimics our natural recall of motor skills. Especially in light of the mixed evidence for a stationary neuron-behavior relationship in cortical motor areas, understanding this relationship during long-term neuroprosthetic control can elucidate principles of neural plasticity as well as improve prosthetic function. Here, we paired stable recordings from ensembles of primary motor cortex neurons in macaque monkeys with a constant decoder that transforms neural activity to prosthetic movements. Proficient control was closely linked to the emergence of a surprisingly stable pattern of ensemble activity, indicating that the motor cortex can consolidate a neural representation for prosthetic control in the presence of a constant decoder. The importance of such a cortical map was evident in that small perturbations to either the size of the neural ensemble or to the decoder could reversibly disrupt function. Moreover, once a cortical map became consolidated, a second map could be learned and stored. Thus, long-term use of a neuroprosthetic device is associated with the formation of a cortical map for prosthetic function that is stable across time, readily recalled, resistant to interference, and resembles a putative memory engram.

## Abstract 2

In this article, the authors show that the neural representation for control of a neuroprosthetic device undergoes a process of consolidation, after which it is stable, readily recalled, and resistant to interference.

## Author Summary

Brain–machine interfaces (BMIs) have the potential to revolutionize the care of neurologically impaired patients. Numerous studies have now shown the feasibility of direct “brain control” of a neuroprosthetic device, yet it remains unclear whether the neural representation for prosthetic control can become consolidated and remain stable over time. This question is especially intriguing given the evidence demonstrating that the neural representation for natural movements can be unstable: BMIs provide a window into the plasticity of cortical circuits in awake-behaving subjects. Here, we show that long-term neuroprosthetic control leads to the formation of a remarkably stable cortical map. Interestingly, this map has the putative attributes of a memory trace, namely, it is stable across time, readily recalled, and resistant to the storage of a second map. The demonstration of such a cortical map for prosthetic control indicates that neuroprosthetic devices could eventually be controlled through the effortless recall of motor memory in a manner that mimics natural skill acquisition and motor control.

## Introduction

Research into the development of brain–machine interfaces (BMIs) [1] has flourished in the last decade, with impressive demonstrations of rodents, nonhuman primates, and humans controlling robots or computer cursors in real time [2]–[17]. Studies of closed-loop cortical BMIs have further demonstrated that improvements in performance require learning [3]–[7],[11],[12],[14]–[17]. Basic research into the neural basis of such adaptations has indicated that changes in the directional tuning properties of neurons are associated with the process of learning [5],[6],[14],[17].

However, the neural plasticity and the cortical dynamics associated with long-term BMI use remains unclear. Studies into the neural plasticity associated with BMI use typically incorporated variable ensembles of neurons from day to day [3]–[7],[11],[12],[14]–[17]. In addition, the transform of cortical activity into a prosthetic motor output (i.e., the decoder) was modified at the start of each daily session. Under such conditions, it is likely that novel neural adaptations were required each day to learn the new transform between neural activity and neuroprosthetic control [5],[6],[12],[16]–[18]. Thus, it remains unclear whether a neural representation for prosthetic function can be stabilized and recalled in a manner that mimics our natural ability to recall motor skills.

A better understanding of the cortical dynamics during long-term neuroprosthetic use is important, both from a basic neuroscience point of view as well as from the perspective of neuroprosthetics. Past studies of the neural basis of natural motor control have presented conflicting evidence for a stable neuron-behavior relationship in motor areas [19]–[25]. For example, whereas some studies have found that the neuron-behavior relationship in primary motor cortex (M1) is constant during stereotyped movements [24], others have shown that this relationship can be nonstationary [20],[23]. Specifically, it remains unclear whether the directional tuning properties of M1 neurons are truly stable across time. It would also be valuable to understand these dynamics during long-term neuroprosthetic control. For example, in the scenario that the neural code for prosthetic control is inherently unstable across time, sophisticated adaptive algorithms may be necessary for long-term reliable performance [21],[25].

To fully delineate the ensemble cortical dynamics during the process of learning and reliably using a BMI, we specifically paired a fixed decoder with stable recordings from ensembles of neurons in two macaque monkeys across a period of up to 19 d. The incorporation of a stable ensemble of putative single neurons across days allows us to track specific changes in neural properties over time. Moreover, as we are primarily interested in understanding the long-term neural adaptations to a fixed transform of neural activity into cursor movements, the decoder was held constant over the time period of each experiment. Using such conditions, we demonstrate for the first time, to our knowledge, the long-term reorganization of motor cortex activity associated with daily practice of a center-out task under brain control. We found that the motor cortex is able to form and consolidate an ensemble cortical map for prosthetic control. This neural representation was found to be remarkably stable across time and could be readily recalled at the start of a daily session.

## Results

### Stability of Recorded Neural Ensembles

**Figure 1:**
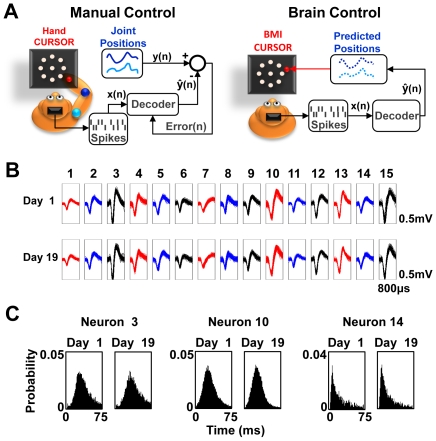
Experimental setup and stability of ensemble recordings. (A) Schematics for manual control (MC) and brain control (BC). During MC, the animal physically performs a two-dimensional center-out task using the right upper extremity while the neural activity is recorded. Under BC, the animal performs a similar center-out task using a computer cursor under direct neural control through a decoder trained during MC. (B) Stability of putative single units across multiple days. Upper panels show a set of waveforms on day 1 versus day 19. The height and width of each box are 0.5 mV and 800 µs, respectively. (C) Stability of firing properties across time. Interspike interval (ISI) distributions are shown for days 1 and 19 for three representative units. There were no significant differences between each pair of distributions (*p*>0.05, Kolmogrov-Smirnov Test).

Two macaque monkeys were first trained to manually perform delayed center-out reaching movements using a robotic exoskeleton that limited movements to the horizontal plane (i.e., manual control, MC). This commercially available robotic system allows precise and accurate measurement of kinematic parameters [26]. Following implantation of microelectrode arrays in bilateral primary motor cortex (M1) (128 microelectrodes in each of the two monkeys), each animal was trained to perform the same center-out task in brain control (BC), in which the neural activity directly controlled the position of the cursor (Figure 1A). In each animal, we could record approximately 75–100 well-isolated units during each daily session. However, consistent with reports in the literature [15],[19],[24],[27]–[31], several months postimplantation, a small ensemble of units were found to be extremely stable across a period of days to weeks. Past studies have demonstrated that ensembles of a similar size can be successfully used for two- or three-dimensional control of neuroprosthetic devices [4],[5].

In the specific experiments presented here, we ensured that the ensemble of neurons used for BC were stable over the time frame of the experiment (hereafter referred to as a “stable neural ensemble”). Stability of well-isolated units across days was first assessed by the stationarity and quality of waveforms (Figure 1B). In order to also quantify the stability of waveforms, we compared waveform characteristics across multiple days using principal components analysis (see Figure S1). Recent studies have indicated that this is a valid metric of waveform stability across days [27]–[31]. As an additional measure, we also ensured that the firing statistics (i.e., interspike interval [ISI] distribution) of each putatively stable single unit did not significantly change from day to day [31]. Figure 1C shows three representative ISI distributions for three single units for two separate days. There were no significant changes in the distributions (*p*>0.05, Kolmogrov-Smirnov Test). Finally, as a measure of ensemble stability across time, we periodically measured the directional tuning of each unit during daily MC sessions. As shown in Figure S2, the ensemble tuning properties were also stable across time.

### Brain Control Performance with Stable Ensembles

In this study, we were primarily interested in understanding the long-term neural adaptations to a fixed transform of neural activity into cursor movements (i.e., a fixed decoder across days). As in previous closed-loop BMI studies [4],[6],[11],[14], we used a linear decoder optimized for physical movements of the upper limb. The linear decoder [6],[21],[24] remains a straightforward and transparent method to transform neural activity into a control signal for closed-loop BMI experiments. As shown in Figure 1A, while the animal physically performed center-out movements during MC, the recorded M1 spike activity was regressed against the elbow and shoulder angular positions to generate correlations for each variable. We will use the term *decoder* to refer to the combined transforms for both shoulder and elbow position. In BC mode (Figure 1A), this decoder allowed neural activity to control the computer cursor. For the initial set of experiments, BC performance was measured in the setting of (1) recordings from a stable ensemble of primary motor cortex (M1) neurons over days, and( 2) a linear decoder that was held constant after training during the MC session on day 1 (hereafter referred to as “fixed decoder”).

**Figure 2:**
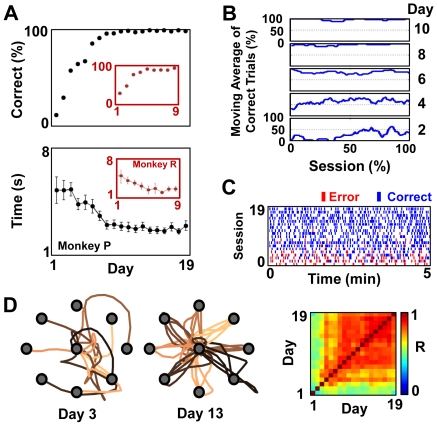
Brain control performance with practice. (A) Changes in BC performance for consecutive days in the setting of a fixed decoder and a fixed set of units in two monkeys (inset = Monkey R). The top panel shows the mean accuracy per day, and the bottom panel shows the mean time to reach each target with training. Error bars represent ±2 standard errors of the mean (s.e.m.). (B) Trends of performance for select days for Monkey P. For each day, the moving average (i.e., percentage of correct trials for a moving window of 20 trials) of performance is shown. (C) Performance during the first 5 min of BC for each daily session. Each bar represents an initiated trial (red = error trial, blue = correct trial). (D) Left: representative examples of single-trial cursor trajectories during the initial (day 3, *n* = 1/target) and the late (day 13, *n* = 5/target) phases of BC performance. The targets are shown in grey. Right: color map of the comparison of mean trajectories for each day. Each pixel represents the pairwise correlation between the mean paths from the center to each of the eight targets. *R* = correlation coefficient.

Figure 2A quantifies the daily performance of the center-out task in BC for two animals with a fixed decoder. Previous studies have used a variety of tasks to study BC. Because these tasks range from discrete to continuous control, it is difficult to directly compare task performance across studies [3]–[17]. In this study, the cursor was under constant neural control, and the subject was required to perform multiple steps for a correct trial (including initiation by movement to the center followed by a brief hold period). Previous studies suggest that such continuous-control, multistep tasks are significantly more difficult than single-step tasks [6],[12]. Accordingly, longer periods of practice were initially required to learn this multistep task in BC. For the experiments from Monkey “P” and “R” shown in Figure 2, ensembles of 15 units and ten units were used, respectively. For both subjects, with daily practice with a fixed decoder, there was a monotonic increase in BC performance and accuracy (Figure 2A).

As also evident in Figure 2A, there was a similar monotonic decrease in the mean time to reach targets. Whereas the initial cursor trajectories meandered, they became more direct with practice (Figure 2D, comparison of representative trajectories from day 3 and day 13 for Monkey P). It is important to note that the subjects were not required to follow a straight path from the center to each target. Interestingly, the mean trajectory to each target became increasingly stereotyped over time, suggesting that a relatively stable solution emerged for the path to each target. We quantified the similarity between each set of daily mean trajectories by performing pairwise correlations (see Materials and Methods). As illustrated by the color map in Figure 2D, the correlation between the mean paths for each day initially increased and then stabilized. Similar results were obtained for Monkey R (see Figure S3)

### Daily Rapid Recall of Performance

We conducted a detailed examination of the performance during each daily session to identify whether BC “skill” could be transferred from one day to the next with practice under these conditions. Past studies have typically presented performance characteristics for an entire session [4]–[7]. As evident in Figure 2B, with practice, subjects could attain accurate performance at the very start of each daily session. Closer examination of the first 5 min of performance each day produced striking evidence of this accuracy at the start of a session (Figure 2C). As expected, there was also a marked reduction in the variability of performance each day under these conditions. Identical levels of performance were also evident in a second animal (Monkey R). Thus, with daily practice in the setting of a stable neural ensemble and a fixed decoder, subjects developed a level of BC skill that could be readily recalled at the start of a session.

### Dynamics of Changes in Ensemble Tuning Properties with Practice

**Figure 3:**
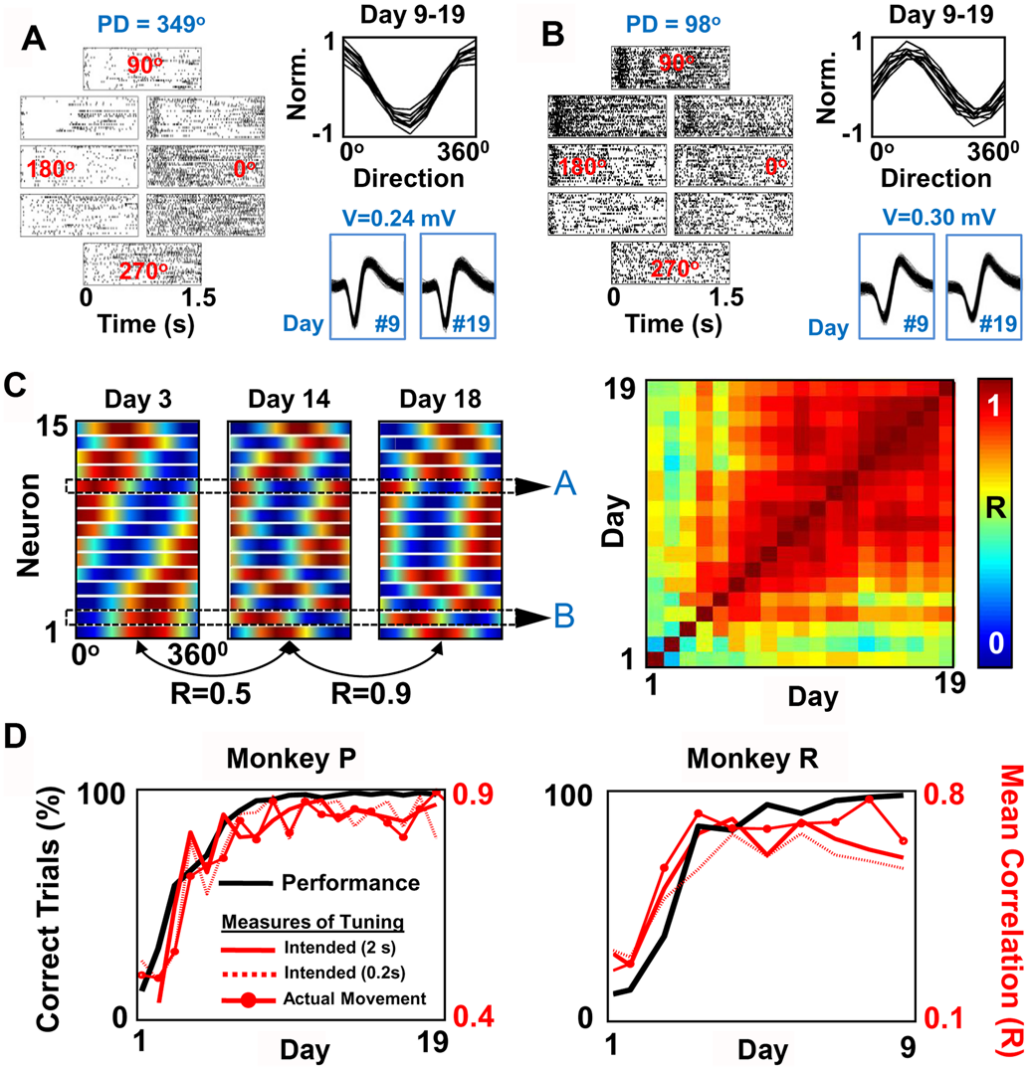
Stable task performance is associated with stabilization of ensemble tuning properties. (A) Tuning properties of a single unit in BC across multiple days. The top panel shows the spiking activity of a neuron during BC (*n* = 26 trials per target). The rasters are arranged to reflect the eight targets in the center-out task (target angles shown in red). Also shown are 200 randomly selected waveforms from two separate sessions (days 9 and 19). The inset shows overlapping tuning curves for each daily session for 10 consecutive days of BC. The shown voltage (V) is the peak-to-peak amplitude for both the shown waveforms. (B) Tuning properties of another unit in BC for ten consecutive days. The panel is arranged similarly to (A). (C) The three color maps to the left illustrate ensemble tuning in BC for days 3, 14, and 18 (specific units from [A] and [B] are labeled accordingly). The units were sorted on day 3 with respect to preferred direction. On the right is a color map of pairwise correlations of ensemble tuning (i.e., map) for each BC session. Warm colors represent a higher level of correlation. (D) Comparison of the learning rate with changes in ensemble tuning for two monkeys. The black solid line reproduces the learning rate from Figure 2. The red solid line represents the average correlation between a daily map and all other ensemble maps (directional tuning was assessed with 2 s of activity relative to intended target. The dotted red line shows the same relationship for directional tuning assessed with a 0.2-s window. The red line with superimposed red dots illustrates the relationship for directional tuning relative to actual cursor movements.

We subsequently characterized the changes in M1 neural activity accompanying the sustained improvements in task performance. For the 19-d experiment shown in Figure 2A, a stable level of performance was evident after day 8. We first examined the neuron-behavior relationship during that period (i.e., days 9 through 19) by calculating the directional modulation of neural activity during BC [32]. The directional modulation of neural activity was initially measured with respect to the intended target. Interestingly, we found that a remarkably stable neuron-behavior relationship was associated with proficient task performance. Figure 3A and 3B illustrate the directional modulation of two representative single units during a single BC session. The insets in Figure 3A and 3B illustrate the stability of this directional tuning relationship for BC across a period of 10 d (no significant changes in preferred direction [PD], bootstrap method, false detection rate [FDR] corrected for multiple comparisons). Overall, 14 of the 15 units did not experience a significant change in PD (bootstrap method, FDR corrected for multiple comparisons). We also evaluated whether this was evident at the level of the neural ensemble. As illustrated by the series of color maps in Figure 3C, we again calculated the daily directional tuning relationship for all units within the ensemble during BC. To compare each daily “ensemble map,” we performed pairwise correlations among each daily set of ensemble tuning properties [6]. The similarity among daily ensemble maps initially increased and then stabilized (Figure 3C).

To compare the temporal course of skill acquisition with the process of map stabilization, we calculated a measure of map similarity across days. Thus, for each day, we calculated the mean correlation for comparisons between a given daily map and all other maps (i.e., mean of each column in the right panel of Figure 3C with exclusion of comparison to self). Remarkably, changes in map similarity closely tracked improvements in task performance for both animals (Figure 3D). Thus, stable task performance was strongly associated with the consolidation of an ensemble activation pattern (a “prosthetic motor map”).

We next examined in greater detail the temporal windows during “movement execution.” For instance, cursor control from the center to each target likely has an initial feedforward stage followed by a period in which visual feedback can lead to path corrections. We thus tested whether a similar stable map emerged when only taking into account the initial stages of execution. As shown in Figure 3D (dotted lines), a similar process of map stabilization also occurred for the first 200 ms of neural activity.

We also performed an additional set of analyses to exclude a potential confounder. As evident in Figure 2D, there was considerable variability in the path taken from the center to each of the targets. It is possible that the apparent evolution of ensemble tuning properties reflects changes in the path as opposed to changes in intrinsic neuronal properties. We thus took into account moment-to-moment changes in the cursor trajectories (i.e., 100-ms steps, see Materials and Methods) when calculating the directional modulation of neural activity (Figure 3D). Unlike the previous analysis based on the intended target, this measure accounts for changes in tuning solely resulting from a modified cursor path. This analysis revealed that the tuning properties of neurons evolved during the period of learning independent of any changes in the actual cursor path.

### Long-Term Changes in the Mean Firing Rate and the Depth of Modulation

The analysis described above focused on changes in preferred direction during learning and long-term use of a neuroprosthetic device. However, past studies have also indicated that other changes in neural properties can also be present [6],[17]. We thus examined the daily changes in the mean firing rate and the depth of modulation of the neural tuning curves. We first compared the mean changes in firing with practice. For Monkey P, eight of 15 units were found to experience long-term changes in the mean firing rate with practice (*p*<0.05. *t*-test comparing days 1–5 with days 15–19, FDR correction for multiple comparisons). Of the eight neurons, seven experienced a net increase, and one demonstrated a slight but significant increase. For Monkey R, six of the ten neurons experienced a significant increase in the mean firing rate with time.

**Figure 4:**
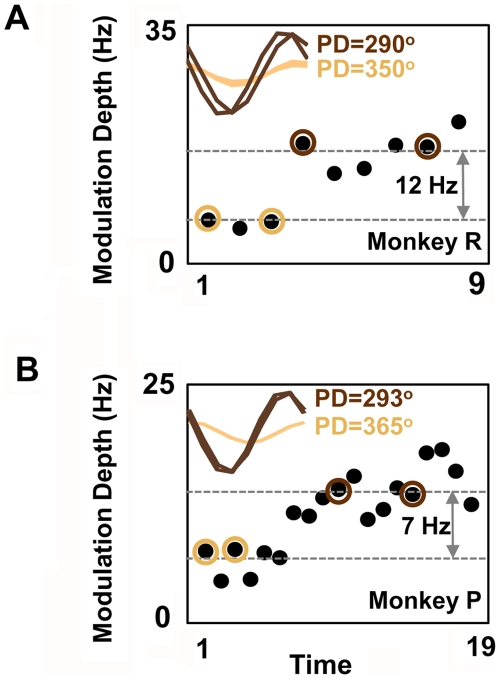
Stable long-term changes in the depth of modulation. (A) Changes in the depth of modulation for a single unit accompanying increases in BC performance (Monkey R). Each dot represents the modulation depth of the neural firing rate (in hertz) for a given BC session. Representative tuning curves from the initial (days 1 and 3 in a lighter color), and late phases (days 4 and 8 in a darker color) of BC are shown in the upper left corner. The shaded circle around each dot identifies sessions represented by the tuning curves. The dotted grey line represents the mean depth of modulation for the respective cluster of dots. (B) Long-term changes in the depth of modulation for a representative single unit from Monkey P, arranged similarly to (A).

We next evaluated for systematic changes in the depth of modulation associated with long-term neuroprosthetic use. Figure 4A and 4B illustrate representative examples of units with a persistent increase in the depth of modulation (*p*<0.05. *t*-test, FDR correction for multiple comparisons). For Monkeys P and R, respectively, seven of 15 and five of ten units demonstrated similar persistent increases in the depth of modulation. The remaining units did not experience significant changes in the depth of modulation. Taken together, these results further highlight the long-term stability of changes in neural properties that tracked improvements in task performance for both animals.

### Importance of the Ensemble Map for Brain Control

**Figure 5:**
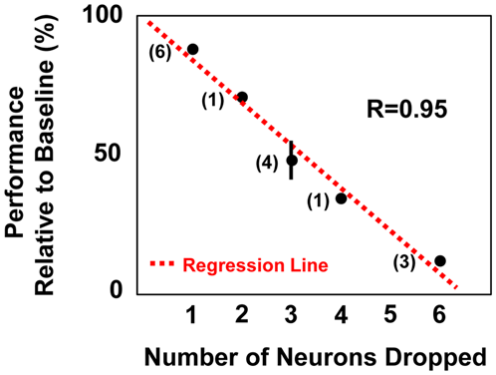
Dependence of BC performance on size of the neural ensemble. Relationship between changes in the neural ensemble size and brain control performance after consolidation of an ensemble map. Plot of changes in BC performance (relative to performance at baseline) after random removal of one to six neurons from the neural ensemble. The error bars represent the standard error of the mean (s.e.m.). For experiments with an *n* = 1, only the mean is shown. For the respective experiments in which only one and six neurons were dropped, the s.e.m. was sufficiently small to be covered by the black dot. Number next to each point represents the number of separate experiments performed. The dotted line represents a linear fit (*R* = 0.95).

Our results thus far suggest that a stable pattern of neural activity is associated with stable BC performance. We next examined whether the entire ensemble is actually involved in BC. For instance, it is possible that only a small fraction of neurons are actually being used for closed-loop BC. We thus generated an “online” neuron dropping curve to quantify the effects of ensemble size on BC performance. After a session in which BC performance was demonstrably accurate (>95% accuracy), a random number of neurons were excluded during subsequent closed-loop BC. Each of these sessions lasted 10 min. We subsequently confirmed that the level of performance returned to the previous baseline. These experiments were performed for both the ten- and the 15-neuron ensembles. As shown in Figure 5, removal of three neurons (i.e., 20% vs. 30% of neurons, depending on the ensemble size) resulted in a greater than 50% drop in accuracy. Moreover, for correct trials under such conditions, it took significantly longer to reach each target (mean time to target of 2.5 s vs. 5.3 s, *p*<0.05, *t*-test). These results indicate that once a neural representation for neuroprosthetic control is consolidated, the entire ensemble map appears to be actively involved in BC.

### Stable Ensemble Map Formation with a Shuffled Decoder

**Figure 6:**
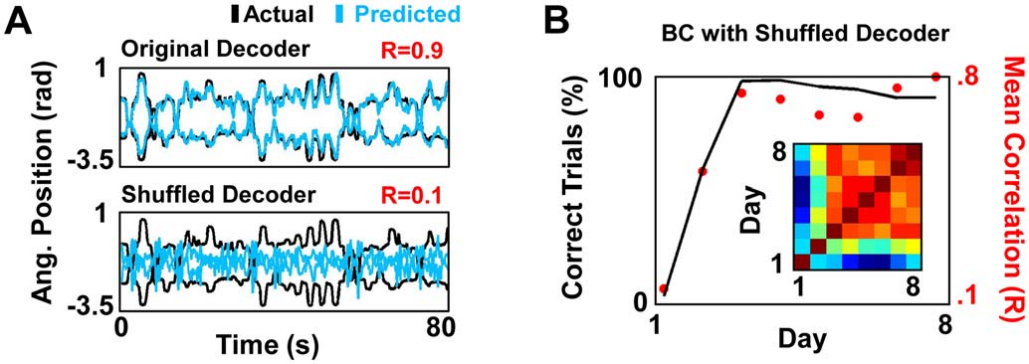
Map stabilization with a shuffled decoder. (A) Comparison of the “offline” predictive ability of an intact and a shuffled decoder. The dark traces are the actual movements. The light blue traces are predictions with each decoder. For each set, the upper trace is the shoulder angular (Ang.) position, and the lower trace is the elbow angular position. *R* is the correlation between the actual and the predicted movements. (B) Temporal course of task performance (solid line) and map stabilization (red dots) for BC with a shuffled decoder. This plot is arranged similarly to Figure 3D. The color map inset shows the pairwise correlation among each daily ensemble tuning map.

Our results suggest that an ensemble of motor cortex neurons can settle upon a remarkably stable activation pattern for prosthetic control in response to a constant decoder. We tested the limits of this conclusion by evaluating whether ensembles of neurons can learn an arbitrary, fixed transform. We thus applied a “shuffled” version of a decoder trained during a MC session. In comparison to the reliable predictions of the actual decoder shown in Figure 6A, the “shuffled decoder” could not reliably predict limb position across time as expected (new ensemble in Monkey P, *n* = 41 neurons). Surprisingly, accurate prosthetic control was achieved after several days of BC practice in the presence of the shuffled decoder (days 3–8: correct trials = 94±1%, mean±standard deviation [SD]; mean time to target = 2.5±0.3 s, mean±SD). Moreover, a stable prosthetic motor map also emerged under these conditions (Figure 6B). In addition to suggesting that a decoder unrelated to arm movements (i.e., a nonbiomimetic decoder) can be learned, this experiment further supports the notion that a stable decoder is crucial for the formation of a stable cortical representation for prosthetic control.

### Specificity of Neural Adaptations

**Figure 7:**
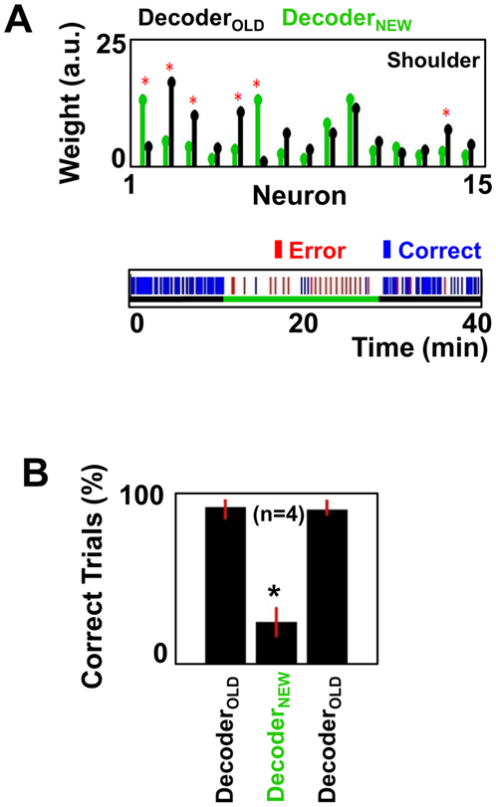
Specificity of neural adaptations to the decoder. (A) Reversible drop in performance with a change in the applied decoder. The upper panel compares the two applied decoders. Pair of bars shows the mean weight for each neuron in each decoder (paired *t*-test, an asterisk [*] indicates *p*<0.05, FDR corrected for multiple comparisons). The lower panel shows the number of correct and incorrect trials in the presence of Decoder_OLD_ (black bar) and Decoder_NEW_ (green bar). a.u., arbitrary units. (B) Changes in performance for similar trials (*n* = 4) in both monkeys with substitution of newly trained decoders (mean±standard error; ANOVA, *p*<10^−5^; the asterisk [*] indicates *p*<0.05, FDR corrected for multiple comparisons).

We subsequently tested the specificity of neural adaptations to the initial fixed decoder. Although many options are available to perturb the transform of neural activity to cursor movements [4],[17], we chose to retrain the linear decoder prior to select BC sessions. The linear decoder was created using multivariate linear regression techniques [33]. It is well known that multivariate linear regression can result in variable model parameters when multiple colinearity is present in the dataset [22],[33]. Thus, two models can be equally effective in predicting a parameter but have different model structures. For prediction of movement parameters from neural data, this can result in slightly different decoder structures (i.e., weight given to each neuron) even while the overall movement prediction is stable [22],[33]. Such variability in the weights can occur for sequential datasets from the same recording session [21],[22],[33]. As shown in Figure 7A (upper panel), similar findings were also evident when two decoders were trained on different days. We thus used daily retraining of the decoder as a means to perturb the transform of neural activity to cursor movements.

Interestingly, substitution of the learned decoder (Decoder_OLD_ in Figure 7A, black bar in upper panel) with a newly trained decoder (Decoder_NEW_, green bar) caused a drop in BC performance. However, the animal could rapidly resume accurate BC upon reinstatement of the well-learned decoder. A significant drop in overall performance was evident for multiple experiments conducted on different days for both animals (Figure 7B). These results suggest that small but significant changes in the model weights are sufficient to prevent an established cortical map from being transformed into a reliable control signal.

We subsequently tested whether a stable prosthetic motor map can emerge in the presence of variability in the decoder. For example, the brain may settle upon a solution that takes into account the inherent variability of the neuron–cursor relationship. We again specifically made use of the variability in the model parameters present with retraining the decoder each day. Under such conditions, more variable daily performance was observed, likely the result of having to relearn the relationship for cursor control each day (see Figure S4A). Moreover, there was no similar trend of cortical map stabilization within the timeframe of the experiment (see Figure S4B). Thus, variability in the decoder impedes the emergence of a stable cortical map for prosthetic control.

### Coexistence of Two Ensemble Maps

**Figure 8:**
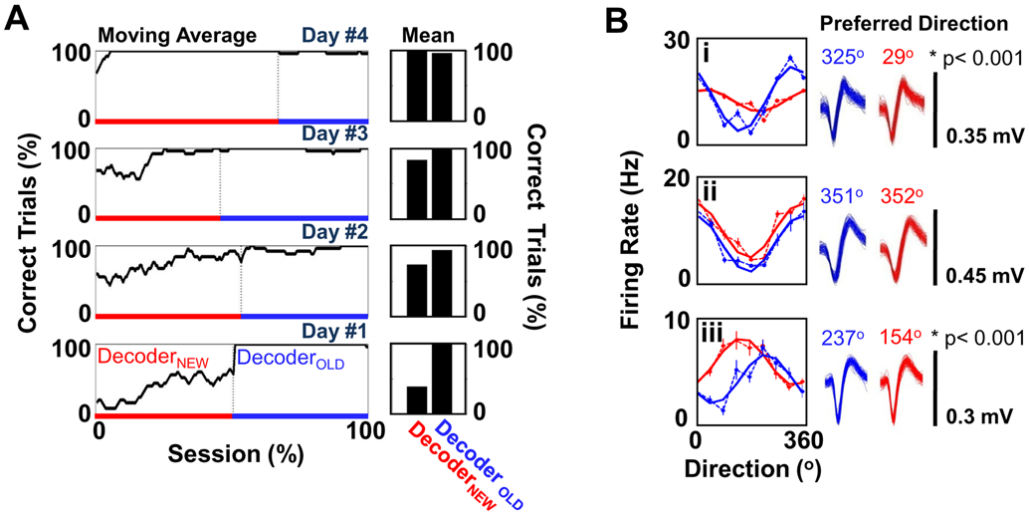
Simultaneous retention of two maps without interference. (A) Changes in performance in the presence of two decoders over 4 d of training. Decoder_NEW_ (red bar) was introduced on day 1. Decoder_OLD_ (blue bar) represents the set of weights that were learned over the course of 19 d of training (as shown in Figures 2 and 3). The panels on the left show a moving average of the performance over the entire session (window size = 20 trials), and the panels on the right represent the mean session performance. (B) Changes in directional tuning for units during BC under Decoder_NEW_ and Decoder_OLD_. Each inset (i–iii) shows the relationship (color convention as in [A]) for the firing rate versus direction (dotted line and filled circles, respectively). The error bars represent the s.e.m. The solid line represents the cosine fit for directional modulation of the firing rate. Shown on the right are 100 randomly selected waveforms for each unit under each of the two conditions. The numbers represent the preferred direction (PD). The asterisk (*) indicates *p*<0.005 for bootstrap analysis with correction of FDR for multiple comparisons.

The results presented above further indicate that the formation of a stable and readily recalled prosthetic map is closely associated with stable task performance. Once stabilized, is a specific prosthetic motor map resistant to interference from learning a second map? To address this question, we examined whether an animal could simultaneously learn and recall cursor control for two distinct biomimetic decoders using the same set of neurons. As shown by our results, a retrained decoder can prevent accurate transformation of neural activity (Figure 7). We thus allowed a subject to practice BC each day using both a “new” biomimetic decoder and a well-consolidated (“old”) biomimetic decoder (Figure 8A). The new decoder was trained during a MC session on day 1. In comparison to the old decoder, there were significant changes in four of the 15 weights (*p*<0.05. *t*-test, FDR correction for multiple comparisons) for the elbow decoder, and seven of the 15 weights for the shoulder decoder (*p*<0.05. *t*-test, FDR correction for multiple comparisons).

As expected, introduction of the newly trained decoder reduced task performance (Figure 8A, day 1). Reintroduction of the consolidated decoder, however, rapidly restored BC performance. Over the course of several days, the subject demonstrated skilled performance with each of the two decoders (day 4, 97.5% vs. 99% trials correct, mean time to target of 2.3 vs. 2.4 s). Surprisingly, the prosthetic motor map was distinct for each of the two decoders. Figure 8B shows examples of changes in directional tuning during BC under each condition (insets i and iii). Nine of 15 units exhibited significant changes in directional tuning (bootstrap, *p*<0.05, FDR corrected). Moreover, although the previously consolidated map remained stable (*n* = 6 comparisons, *R* = 0.86±0.03, mean±SD), the new prosthetic motor map was less similar to previous maps (*n* = 6, *R* = 0.3±0.05, mean±SD).

As suggested previously, these changes in directional tuning could be the result of a change in the cursor path. As the subjects were not required to reach the targets with a straight path, there was some variation between the cursor paths for trials under each of the two decoders (See Figure S5). We next tested whether changes in the path could account for the observed change in directional tuning. We again computed the directional modulation of neural activity with respect to the actual cursor path during the first 200 ms (as opposed to direction of intended movement to a given target). Using this measurement, the calculated PDs were somewhat different for each neuron (compare tuning curves in Figure 8B with those in Figure S6). This likely reflects the difference between the actual curved paths taken in comparison to an idealized straight path (i.e., directional modulation based on the intended direction). As such, there was a systematic shift in the respective PDs for each neuron (e.g., Figure 8B vs. Figure S6: [i] PD_new decoder_ = 29° vs. 96°; [ii] PD_new decoder_ = 352° vs. 74°). Most importantly, even after taking into account the variations in the actual path of the cursor, significant changes in neural tuning were evident during BC with each of the two decoders (see Figure S6).

## Discussion

In summary, this study demonstrates that the motor cortex can form a stable neural representation for neuroprosthetic control. The stability of the emergent cortical map across days is remarkable given that these neurons also participate in the control of natural arm movements for a greater part of the day (in comparison to the approximately 2 h of BC each day). Our results further suggest that the stationarity of this relationship relies upon the constancy of the decoder that transforms neural activity into cursor movements. Interestingly, under such conditions, even nonbiomimetic shuffled decoders allowed the formation of a cortical map that is readily transformed into cursor movements and reliable task performance.

### Formation of a Stable Cortical Map for Neuroprosthetic Control

Our analysis of the neural activity during the period of learning indicates that the neuronal tuning functions (i.e., PDs, mean firing rates, and the depth of modulation) appear to undergo a period of modifications after which a stable ensemble activity pattern emerges. These tuning functions were estimated using either the intended direction of motion (i.e., idealized straight path to the target) or the actual motion of the cursor. For natural motor control, different brain areas may represent each of these aspects [34]. During BC, although these two methods can result in different estimations of neuronal tuning properties (depending of cursor path), they provide complementary estimates of the neuron-behavior relationship during prosthetic control [5],[6]. Together, they indicate that a truly stable neuron-behavior relationship emerges with practice.

The stability of neuronal properties at the level of the ensemble further suggests that a functional cell assembly may have formed during the process of learning and continued daily practice [35]. Accordingly, it is possible that systematic alterations in the dynamics of interneuronal correlations also accompany the long-term modifications of single neuronal tuning properties [36].

A topic for future research is the relationship between feedforward “internal models” and feedback during active neuroprosthetic control [37]. The emergence of stable ensemble activity patterns during the early part of each trial (e.g., the first 200 ms of cursor movement) suggests that BC practice leads to the formation of an internal model for cursor control. It is less likely that visual feedback is responsible for shaping these early time periods [16],[38].

A better understanding of these two factors will elucidate principles of trajectory formation during BC. Interestingly, the emergence of stereotyped trajectories that are not necessarily straight is consistent with a recent study suggesting that the process of motor learning balances the acquisition of reward states with the costs of movement [39]. Under this formulation, optimal paths do not necessarily follow a straight trajectory. Consistent with this concept was also the finding that cursor trajectories under each of the two decoders were both curved and somewhat different for each set of trials.

### Stability of the Neuron-Behavior Relationship

Our findings add to the recent debate on the stability of the neuron-behavioral relationship for both natural motor control and for neuroprosthetic control. Studies have presented conflicting evidence for the notion of a stable neuron-behavior relationship for stereotyped and free-arm movements [19]–[25]. Possible reasons for the apparent variability include the process of learning a novel motor relationship [40]–[42], postural changes, and subtle changes in the pattern of muscle activation [20],[23]. Our experimental setup allowed us to address this in a setting in which the output of an ensemble of neurons can be controlled. Thus, neural activations for a purely disembodied BC task can achieve a stable neuron-behavior relationship after an initial period of instability during learning.

### Comparison to Past Studies

Past studies have presented evidence of long-term improvements in neuroprosthetic control with practice [3]–[7],[11],[12],[14]–[17]. As indicated by our results, however, there are at least two distinct mechanisms for such long-term improvements in performance. There are improvements in learning as a result of the formation and consolidation of a neural representation for prosthetic control. Alternatively, long-term improvements in performance can be the result of daily relearning and the formation of a novel neural representation. Our experiments indicate that incorporation of stable neural ensembles and a fixed transform of neural activity allows for monotonic and reliable improvement in performance. That consolidation of a cortical representation was important for these improvements is suggested by (1) evidence for rapid recall of performance at the start of each daily session, and (2) stabilization of neural tuning functions.

### Choice of Decoding Technique for Neuroprosthetic Control

Our primary interest in this study was to characterize the long-term dynamics of the neuron-behavior relationship for direct cortical control of a cursor. This was best achieved by applying a constant decoder across time while observing the changes in neural activity. Although many decoders are likely to be useful for this purpose, the linear decoder has proven to be effective and offers a ready comparison to past successful BMI studies [4]–[7]. Moreover, our results suggest that cortical map formation can be truly independent of the exact decoder used (e.g., Figure 5 shows learning across days with a shuffled decoder).

One implication of our findings is that cortical control of a prosthetic device depends on specific neural adaptations to the applied decoder [1],[43]. Whereas two decoders may both predict MC movement parameters equally well, there may be significant variability in the parameters assigned to a specific neuron [33]. As shown by our results, this variability prevents the formation of a stable neural representation. Minimizing decoder variability would be less important if an entirely new set of neurons are recorded each day. However, in the more likely scenario where subsets of neurons are stable across time [15],[19],[24],[27]–[31], it will be important to consider parameters assigned to stable units. Taking into account such information could allow “graceful degradation” of function, where the loss of a subset of units would not be catastrophic. This may also minimize the extent of required relearning with changes in the recorded ensemble.

### Role of a Stable Neural Representation for Prosthetic Control

Our results further indicate that the formation and stabilization of a cortical map for prosthetic function is closely linked to the process of long-term neuroprosthetic skill acquisition. Strikingly, the features of this map (i.e., readily recalled, stable, and resistant to interference) resemble properties often attributed to a putative long-term memory engram [44]. It is easy to imagine that in real-world situations, complicated neuroprosthetic control will require consolidation of an analogous “prosthetic motor memory” for long-term retention of skilled function [45]. With continued improvements in technology [46],[47], neuroprosthetic devices could be controlled through effortless recall of such a motor memory in a manner that mimics the natural process of skill acquisition and motor control.

## Materials and Methods

### Surgery

Two adult male rhesus monkeys (*Macaca mulatta*) were chronically implanted in the brain with arrays of 64 Teflon-coated tungsten microelectrodes (35 µm in diameter, 500-µm separation between microwires) in an 8×8 array configuration (CD Neural Engineering). Monkey P was implanted in the arm area of primary motor cortex (M1) and the arm area of dorsal premotor cortex (PMd), both in the left hemisphere, and the arm area of M1 of the right hemisphere, with a total number of 192 microwires across three implants. Monkey R was implanted bilaterally in the arm area of M1 and PMd (256 microwires across four implants). Localization of target areas was performed using stereotactic coordinates from a neuroanatomical atlas of the rhesus brain [48]. Implants were targeted for pyramidal tract neurons in layer 5, and were typically positioned at a depth of 3 mm in M1 and 2.5 mm in PMd. Depth of electrode placement was guided by intraoperative monitoring of spike activity. All procedures were conducted in compliance with the National Institutes of Health *Guide for the Care and Use of Laboratory Animals* and were approved by the University of California at Berkeley Institutional Animal Care and Use Committee.

### Electrophysiology

Unit activity was recorded using the MAP system (Plexon). For this study, only units from primary motor cortex were used. Only single units that had a clearly identified waveform with a signal-to-noise ratio of at least 4∶1 were used. Activity was sorted prior to recording sessions using an on-line spike-sorting application (Sort-Client; Plexon). Large populations of well-isolated units (∼75–100) were recorded during each daily session in both monkeys (typical number of units was defined by waveform quality and ISI distributions). Consistent with reports in the literature [24],[27]–[31], several months postsurgery, we found a subset of stable units whose waveform shape, amplitude, and relationship to other units on a channel varied little from day to day (i.e., the sorting template in the Sort-Client required no or very minor daily modifications). The stationarity of such properties was the first criterion for a putative stable unit. We also examined the properties of the ISI distribution and the presence of an absolute refractory period to confirm the presence of a stable single unit. We also confirmed the stability of the waveforms using commercially available software (Wavetracker; Plexon). Specifically, we utilized the features that allow mapping of waveform characteristics into a two- and three-dimensional principal components space. Stability of waveforms could be assessed by comparison of the stability of the projections across time (please see Figure S1 for examples). Multivariate ANOVA tests allowed statistical comparison.

Moreover, we also estimated the PD in MC of select ensembles of putative stable units. For these subsets of ensembles, MC sessions were performed each day to estimate the directional tuning curves (e.g., Figure S2 shows the similarity of the tuning curves within an ensemble across days). Moreover, the precise number of units per experiment was determined by examining all recorded units over a period of several days to ascertain units with stationary properties. Our conclusions did not appear to depend on ensemble size.

### Experimental Setup and Behavioral Training

Monkeys were trained to perform a center-out delayed reaching task using a Kinarm (BKIN Technologies) exoskeleton. In this device, the shoulder and elbow are restricted to move in the horizontal plane, giving two degrees of freedom (flexion/extension). During training and recording, animals sat in a primate chair that permits limb movements and postural adjustments. Head restraint consisted of the animal's headpost fixated to a primate chair. Recording sessions typically lasted 2–3 h per day. Kinematic variables (position, velocity, and acceleration) were continuously monitored and recorded.

The behavioral task consisted of hand movements from a center target to one of eight peripheral targets (i.e., center-out task) distributed over a 14-cm diameter circle. Target radius was typically 0.75 cm. Trials were initiated by entering the center target and holding for a variable time period of 500–1,000 ms. The “GO” cue (center changed color) was provided after the hold period. A liquid reward was provided after a successful reach to each target and a peripheral hold period (200–500 ms). Visual feedback of hand position was provided by a cursor precisely colocated with the center of the hand (cursor radius = 0.5 cm). During the task, the nontask arm was immobilized in a padded splint.

In BC, the cursor was continuously controlled by neural activity, and each animal received visual feedback of cursor movements. The task-related hand (right) was removed from the exoskeleton and restrained during BC. The cursor was under continuous volitional control throughout the experiment. The subjects were required to self-initiate each trial by bringing the cursor to the center. As mentioned below, the hold period for BC was optimized in order to minimize false-positive activations.

Typical BC trials required a fixed center hold period of 250–300 ms. As in other studies [6], subjects experienced difficulty completely stopping the cursor. During typical hold periods, the cursor slowed down enough to trigger the GO cue. However, with practice (e.g., after >6–7 d for a given set of neurons and a fixed decoder), animals could perform tasks that required longer hold periods (e.g., 1,000 ms) as well as variable hold periods. During these trials, the cursor appeared to be actively held in place. Moreover, reward was provided when the cursor was inside of the peripheral target for >100 ms. Typically, a reduction in velocity was sufficient to accomplish this.

A trial was considered incorrect if the cursor failed to reach the target within 10 s after a GO cue. During selected sessions, we concurrently performed video and surface electromyelogram (EMG) recordings from proximal muscle groups. As in past studies, neither animal moved their upper extremity during BC [5],[6]. The observation that movement was not critical for BMI performance is further highlighted by the fact that a shuffled decoder with no relation to actual movements could be learned.

During experiments in which new decoders were introduced (e.g., see Figure 7), no cues were given. These blocks occurred in a randomized, unpredictable manner. Moreover, these trials were brief (∼20 min). However, for experiments in which two decoders needed to be learned, two different color-coding schemes were used to indicate differences between BC sessions involving the two decoders (e.g., data shown in Figure 8). For these experiments, the color of peripheral targets was different for trials using either the old decoder (blue) or the new decoder (yellow). The respective color schemes for the center target (green) and the GO cue (change from green to red) remained constant. In experiments requiring relearning of a daily decoder (i.e., data shown in Figure 4), animals were given longer sessions (1–2 h) in order to adapt to the changes.

Finally, there was evidence of generalization of prosthetic control beyond the stereotyped structure of the center-out task. In selected experimental blocks, animals were able to generate novel cursor trajectories in order to reach the targets (see Figure S7).

### Decoding Motor Parameters from Neural Ensembles

Previous analyses [2],[6],[21] have demonstrated that hand position and velocity can be accurately predicted with a linear regression model. In this model (Equation 1), the inputs, *X(t)*, were a matrix with each column corresponding to the discharges of individual neurons, and each row representing one time bin. The output *Y*(*t*), was a matrix with one column per motor parameter. The linear relationship between neuronal discharges in X(*t*), and behavior (elbow and shoulder joint positions) in *Y*(*t*) was expressed as
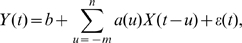
(1)where **a** and **b** are constants, calculated to fit the model optimally. First, a(*u*) are the impulse response functions required for fitting *X*(*t*) to *Y*(*t*) as a function of time lag *u* between the inputs and the outputs. Ten time lags were used during these experiments. Second, b represents the *Y*-intercept in the regression. The final term in the equation, *ε*(*t*), represents residual errors. The linear filter was generated using the techniques described above and neural (spike activity from a select group of neurons binned into 100-ms bins) and kinematic data (continuous recordings of the elbow flexion/extension and shoulder flexion/extension angles) recorded from a 10-min session of MC (while performing the center-out task). Past studies have shown that a bin size of 100 ms is optimal [2],[6],[21]. A new decoder was trained by repeating the algorithm outlined above during a MC session on subsequent day.

#### Shuffled decoder

The shuffled decoder was generated by shuffling the exact relationship between the neurons used for training and predicting. Thus, after training a new decoder (ten lags/neuron), a randomized shuffle was performed such that each set of ten lags was randomly assigned to a neuron. As shown in Figure 5, this dramatically reduced the ability to predict limb position over time.

It should be noted that unlike in other studies, we initially created decoders that predicted joint kinematics as opposed to hand-centered kinematics. For our M1 recording from both animals, we found that predictions of joint kinematics were more reliable than hand-centered kinematics. Thus, we initially attempted to maximize the ability of the linear decoder to predict manual control trajectories by first predicting joint kinematics. However, as indicated by the experiments with the shuffled decoder, a clear relationship between the decoder and MC was ultimately not found to be essential for accurate BC.

Past studies have successfully used both position [14] and velocity [5],[6] control for BC. A likely difference between position control and velocity control is that hold periods at different locations in the workspace require different patterns of activity. For example, whereas returning to a single state allows for holding at any location for velocity control, position control requires different states for varying locations in the workspace.

#### Brain–machine interface

We used the linear filter described in the previous section to predict shoulder and elbow joint angles from the recorded neural activity. The model was trained on 10 min of activity and then used to predict position from subsequent neural activity. Filter parameters were not changed during the BC experiments. Neural activity was streamed over a local intranet via the PLEXNET client-server application (Plexon) and converted into 100-ms bins of spiking activity. Each binned value was used to generate real-time predictions of the shoulder and elbow joint angles that were streamed to the Kinarm interface as control signals. These predictions were converted into Cartesian coordinates (i.e., *xy* position of the cursor) through a Jacobian matrix. The cursor position was updated on the Kinarm projection screen at 10 Hz.

### Data Analysis

#### Task performance analysis

The BC task was calibrated to minimize false positives for “self-initiation” and “correct trials.” To start a trial, the cursor had to be held over the center target for 250–300 ms. The chance level of self-initiation was approximately 0.5 trials per minute. This value was determined through experiments in which the task was performed by spontaneous neural activity (i.e., the computer monitor was turned off while the cursor was controlled by spontaneous activity). In contrast, while engaged in the task, each subject self-initiated trials at a rate of 3–10/min. The lower end of the range was seen during unskilled BMI performance (Figure 2C). A false-positive correct trial (self-initiation followed by target acquisition) was rare (typically ∼one per 10 min).

Both trial attempts and correct trials were counted from the instant BC was initiated each day. A correct trial was defined as successful movement of the cursor to the target followed by the hold period. As indicated above, we minimized the number of false-positive self-initiations (i.e., the number of trial attempts). The time-to-target measurement (Figure 2B) reflected the movement time from the center to each peripheral target.

#### Predictive power of the decoder

The predictive power of each decoder was determined by comparison (i.e., correlation) of neural predictions of shoulder and elbow angular position with that of measured values. Estimation of predictive power was performed using 2 min of movements outside of the 10-min training window. As in past studies [2],[6], there was a positive relationship between predictive power and the size of a randomly selected neural ensemble. However, our conclusions did not vary with respect to the size of a neural ensemble. Thus, the factors enabling skill acquisition were identical for both the larger (*n* = 41) and the smaller (*n* = 10–15) ensemble sizes.

#### Preferred direction

Directional tuning was estimated by comparing the mean firing rate as a function of target angle during execution of the movement [24],[32]. In MC, the time to target was fairly constant. In BC, however, this time period was variable and often decreased with stabilization of prosthetic skill. We thus calculated the mean time to target for the entire experimental set (e.g., over the 19 d shown in Figure 2, mean time to target was 2.3 s). We subsequently used the time period of 2 s as the window for calculating the mean firing rate versus target direction relationship for subsequent experiments. The first 2 s of each trial were used. A similar method was also used for shorter time windows (e.g., 200 ms). Essentially identical results were obtained with window sizes of 1 s and 1.5 s (e.g., see evolution of spiking activity in raster plot in Figure 3A and 3B). The tuning curve was estimated by fitting the firing rate with a sine and a cosine as:
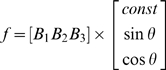
(2)where *θ* corresponds to reach angle and *f* corresponds to the firing rate across the different angles. Linear regression was used to estimate the *B* coefficients. The PD was calculated using the following: PD = tan^−1^ (*B_2_/B_3_*), resolved to the correct quadrant. The depth of modulation was measured by calculating the difference between the maximum and the minimum of the fit curve. *B1* was taken to be mean firing rate for a session. The depth of modulation was measured by calculating the difference between the maximum and the minimum of the tuning curve (in hertz). *B1* was taken to be mean firing rate for a session.

For the analysis of the directional modulation of the firing rate with respect to the actual direction of cursor movements, movement direction was measured every 100 ms. For each neuron, the firing rate was calculated for the preceding 100-ms interval. Directions were binned into 16 bins (i.e., a bin size of 22.5° for the range of 0° to 360°). The respective neural firing rate was then determined for each bin. For the analysis shown in Figure 3D and Figure S6, the first two steps (i.e., 200 ms total) were used to estimate the tuning curves. The tuning curve was estimated using linear regression as outlined earlier.

#### Changes in directional tuning of neuron

A bootstrap resampling procedure was used to assess the statistical significance of preferred direction changes [24]. The bootstrap statistics involved the following steps: (1) for a given session and unit, a distribution of PDs was generated by bootstrap sampling with replacement of the observed unit's spiking activity versus target direction; (2) a cosine tuning model was generated for each sampling; and (3) the circular mean was subtracted from each PD. For comparison between two units, we sampled one PD from each zero mean distribution to create a distribution of absolute angle difference. By repeating this procedure 2,000 times, we created a distribution corresponding to the null hypothesis (no change in PD). This distribution was used to compute the probability that the actually observed change in PD was statistically zero. Units whose PD difference had a *p*-value 0.05 corrected for multiple comparisons (i.e., false detection rate [FDR] with a Bonferroni-type correction) were considered to have a significant change in PD.

#### Index of similarity between ensemble tuning maps

We used pairwise correlation among the ensemble tuning maps (both for MC and for BC) to assess similarity between two maps [6]. In Figure 3A, 3B, 3C, and Figure S4A, the neural tuning curves were normalized to the peak positive value for a given day, and constant term was not included. This readily allowed for comparison of changes in the preferred direction for the entire ensemble over time (e.g., Figure 3C and Figure S4B). For the comparison of the map similarity with respect to the learning curve for task performance (e.g., Figure 3D), we calculated the mean correlation between all the maps across days (with exclusion of the self-comparison). Thus, each point on the curve shown in Figure 3D (left panel) represents the average of 18 values.

#### Index of similarity between BC trajectories

We also used pairwise correlation between the mean trajectories to each target per day to assess whether a more stereotyped trajectory was present with practice. For each daily session, the mean path to each target was calculated by averaging all correct trials. A mean correlation value (for a given comparison between two days) was obtained by averaging the correlation between the sets of paths.

## Supporting Information

Figure S1 **Stability of spike waveforms over time.** (A) The panels on the left show samples of 100 randomly selected waveforms from a single channel on days 1, 5, 10, and 15. The width and height of each box are identical. The panel on the right shows the mapping of the waveforms from every other day (from days 1 to 15) onto a two-dimensional principal component (PC) space (i.e., “waveform stability tube”). The z-axis represents time (in days). Each of the distributions are also shown in an overlapping manner below the tube (PC1 vs. PC2 axis). (B) Another example of waveforms from multiple days and the corresponding “waveform stability tube.” The panels are as in (A).
(0.26 MB TIF)

Figure S2 **Stability of directional tuning in manual control.** (A) The group of panels illustrates the properties of nine units from the 41-unit ensemble shown in Figure 6 (Monkey P). Each set of panels shows the waveform for a unit (day 4) and eight overlapping mean tuning curves for that unit during manual control (days 1 through 8). Each curve represents a cosine fit to the directional modulation of the firing rate. There were no significant changes in the PD (bootstrap, FDR corrected for multiple comparisons). (B) Examples of the path taken from the center to each of the eight targets during performance of manual control trials. (C) Pairwise correlation of the MC ensemble tuning map across 8 d. Arranged similarly to Figure 3C.
(0.75 MB TIF)

Figure S3 **Comparison of daily mean cursor trajectories.** Representative cursor trajectories from an early (*n* = 1/target) and late session (*n* = 5/target). The color map on the right represents a pairwise correlation of mean trajectories to all targets per day for Monkey R. Warm colors represent higher correlations than cooler colors. Thus, the mean trajectories become increasingly stereotyped after attaining a stable performance level (e.g., after day 3).
(0.12 MB TIF)

Figure S4 **Variations in the ensemble tuning map using a new daily decoder.** (A) For the 3 d shown are representative waveforms, performance characteristics, and the ensemble tuning map from the BC session. The performance data represent BC after a period of adaptation to the change in decoder properties. With a new daily decoder, there was substantial variability in the neuronal directional tuning for BC each day. This indicated that the motor cortex had to form a new cortical map to successfully translate neural activity into cursor movements. The color maps represent data used to calculate the pairwise correlation map shown in (B). (B) Color map of the pairwise correlations of the ensemble tuning during BC with daily retraining of the decoder. Each session represents BC performed under a newly trained decoder. This figure is arranged similarly to Figure 3C.
(1.14 MB TIF)

Figure S5 **Single-trial cursor trajectories for BC under two decoders.** Examples of the single-trial cursor paths during experiments in which two decoders had been simultaneously learned. These represent the neural data analyzed in Figure 8. Each panel shows the cursor path from the center to a target (*n* = 5). The green circles indicate the active target for a given panel.
(0.23 MB TIF)

Figure S6 **Changes in directional tuning measured with respect to the path of cursor movements.** Comparison of the neural tuning functions during BC with two different decoders. The three panels represent a separate analysis of the neural data presented in Figure 8B. Although the tuning functions shown in Figure 8 represent the directional modulation of the neural activity relative to the intended target, the tuning functions shown here represent direction modulation with respect to the actual path of the cursor (first 200 ms). Thus, even after taking into account changes in the path of the cursor, there were changes in the tuning functions. The color scheme is identical to that used in Figure 8.
(0.13 MB TIF)

Figure S7 **Generalization of prosthetic control beyond the center-out task.** Sample trials demonstrating generalization of prosthetic control beyond the stereotyped center-out task. The figure shows how Monkey P can control the cursor from an arbitrary start target. The original task was altered such that the “Start” target (shown in red) randomly appeared within the workspace in an unpredictable pseudorandom order. Although the “End” target (green) remained as prior, novel unrehearsed trajectories were required to reach each of the eight targets. The accuracy level for this new task was typically >85% and did not appear to require extensive relearning.
(0.09 MB TIF)

## Abbreviations

BC: brain control
BMI: brain–machine interface
FDR: false detection rate
ISI: interspike interval
MC: manual control
PD: preferred direction
