# Supplementary figures and images for: Emergence of a Stable Cortical Map for Neuroprosthetic Control
Source: PLoS Biol. 2009 Jul 21;7(7):e1000153. doi: 10.1371/journal.pbio.1000153 (PMC2702684; doi:10.1371/journal.pbio.1000153)

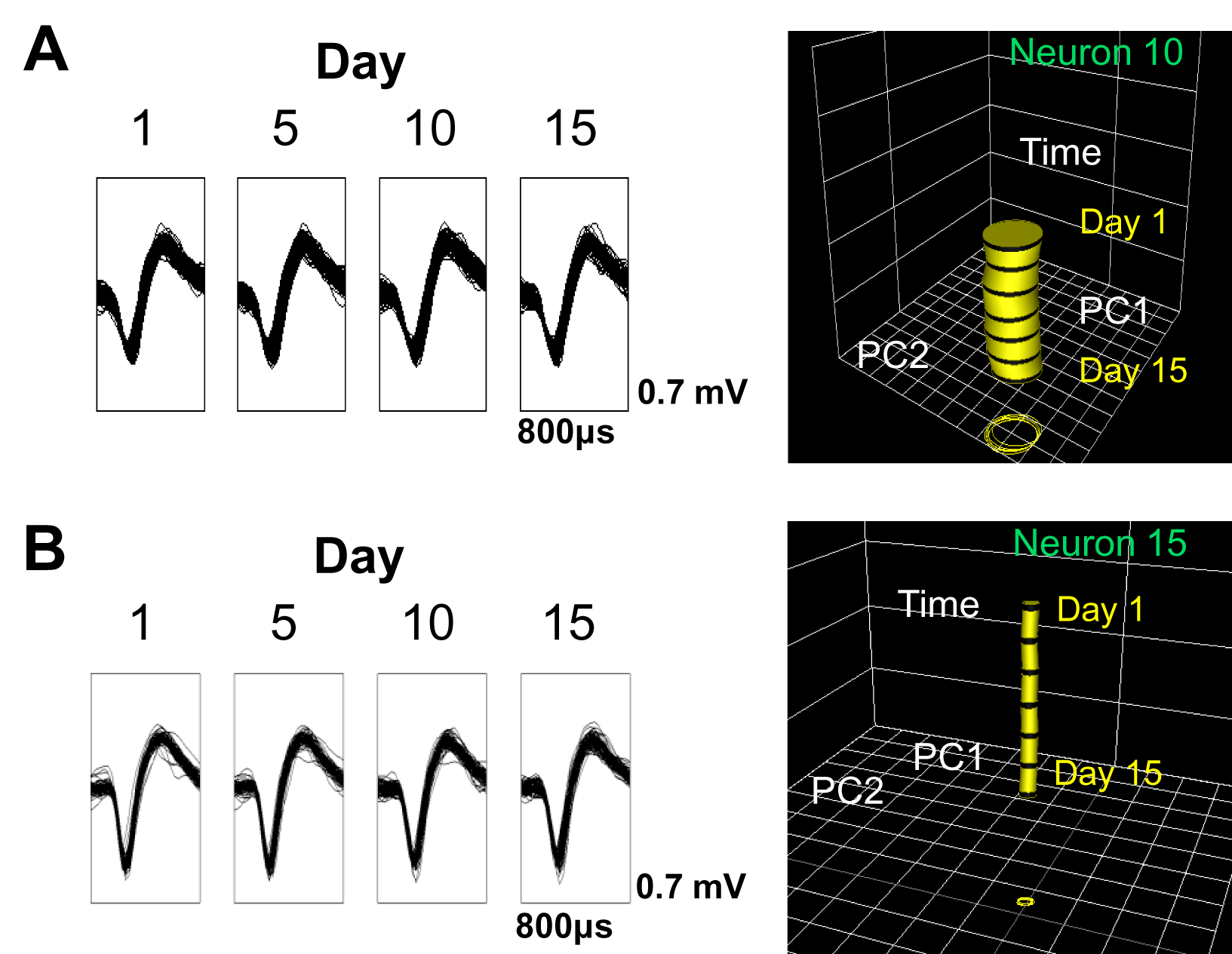

Supplement: Figure S1 — Stability of spike waveforms over time. (A) The panels on the left show samples of 100 randomly selected waveforms from a single channel on days 1, 5, 10, and 15. The width and height of each box are identical. The panel on the right shows the mapping of the waveforms from every other day (from days 1 to 15) onto a two-dimensional principal component (PC) space (i.e., “waveform stability tube”). The z-axis represents time (in days). Each of the distributions are also shown in an overlapping manner below the tube (PC1 vs. PC2 axis). (B) Another example of waveforms from multiple days and the corresponding “waveform stability tube.” The panels are as in (A). (0.26 MB TIF) [file pbio.1000153.s001.tif]

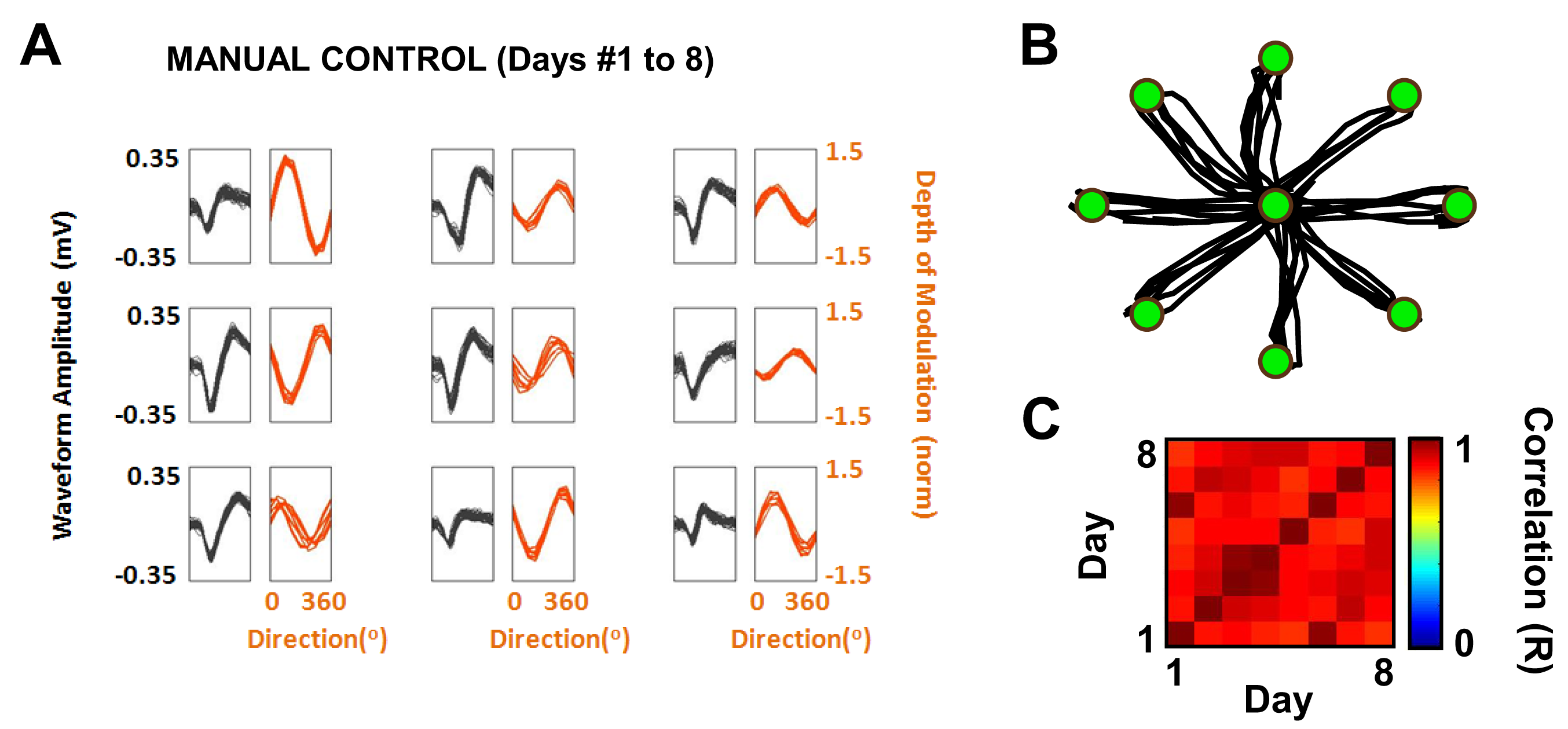

Supplement: Figure S2 — Stability of directional tuning in manual control. (A) The group of panels illustrates the properties of nine units from the 41-unit ensemble shown in Figure 6 (Monkey P). Each set of panels shows the waveform for a unit (day 4) and eight overlapping mean tuning curves for that unit during manual control (days 1 through 8). Each curve represents a cosine fit to the directional modulation of the firing rate. There were no significant changes in the PD (bootstrap, FDR corrected for multiple comparisons). (B) Examples of the path taken from the center to each of the eight targets during performance of manual control trials. (C) Pairwise correlation of the MC ensemble tuning map across 8 d. Arranged similarly to Figure 3C. (0.75 MB TIF) [file pbio.1000153.s002.tif]

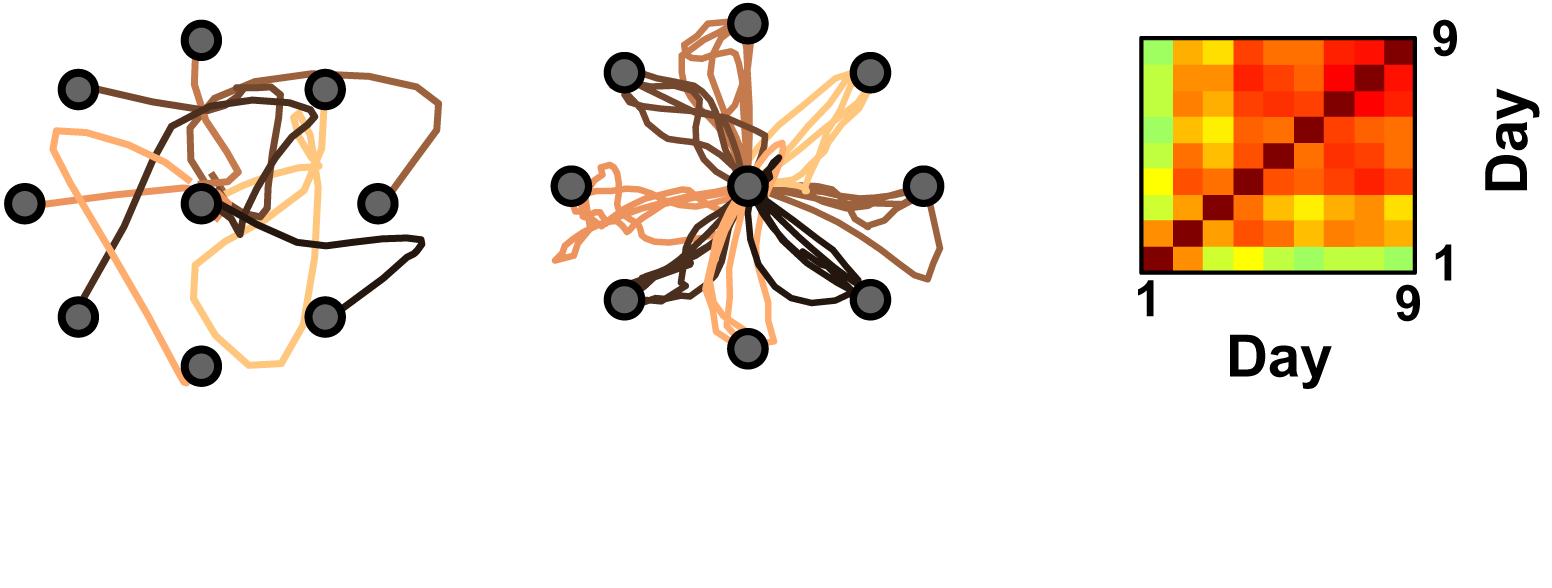

Supplement: Figure S3 — Comparison of daily mean cursor trajectories. Representative cursor trajectories from an early (n = 1/target) and late session (n = 5/target). The color map on the right represents a pairwise correlation of mean trajectories to all targets per day for Monkey R. Warm colors represent higher correlations than cooler colors. Thus, the mean trajectories become increasingly stereotyped after attaining a stable performance level (e.g., after day 3). (0.12 MB TIF) [file pbio.1000153.s003.tif]

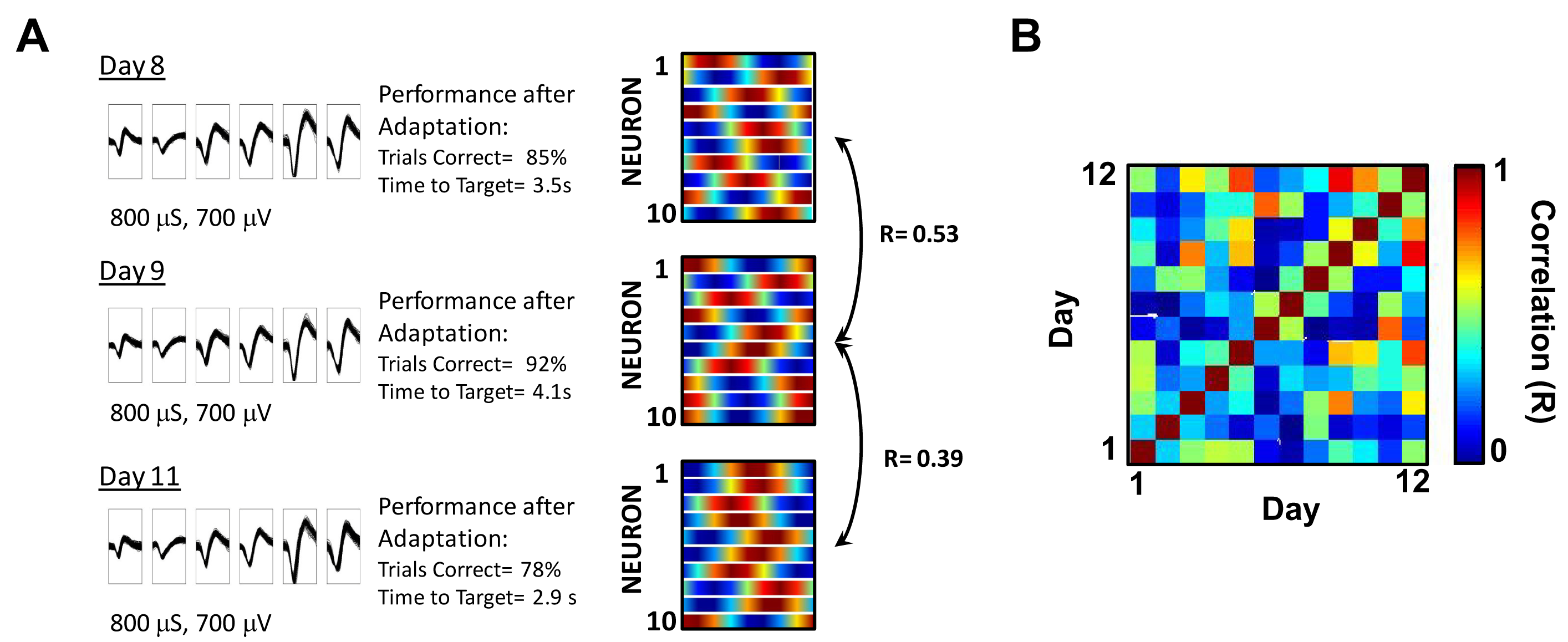

Supplement: Figure S4 — Variations in the ensemble tuning map using a new daily decoder. (A) For the 3 d shown are representative waveforms, performance characteristics, and the ensemble tuning map from the BC session. The performance data represent BC after a period of adaptation to the change in decoder properties. With a new daily decoder, there was substantial variability in the neuronal directional tuning for BC each day. This indicated that the motor cortex had to form a new cortical map to successfully translate neural activity into cursor movements. The color maps represent data used to calculate the pairwise correlation map shown in (B). (B) Color map of the pairwise correlations of the ensemble tuning during BC with daily retraining of the decoder. Each session represents BC performed under a newly trained decoder. This figure is arranged similarly to Figure 3C. (1.14 MB TIF) [file pbio.1000153.s004.tif]

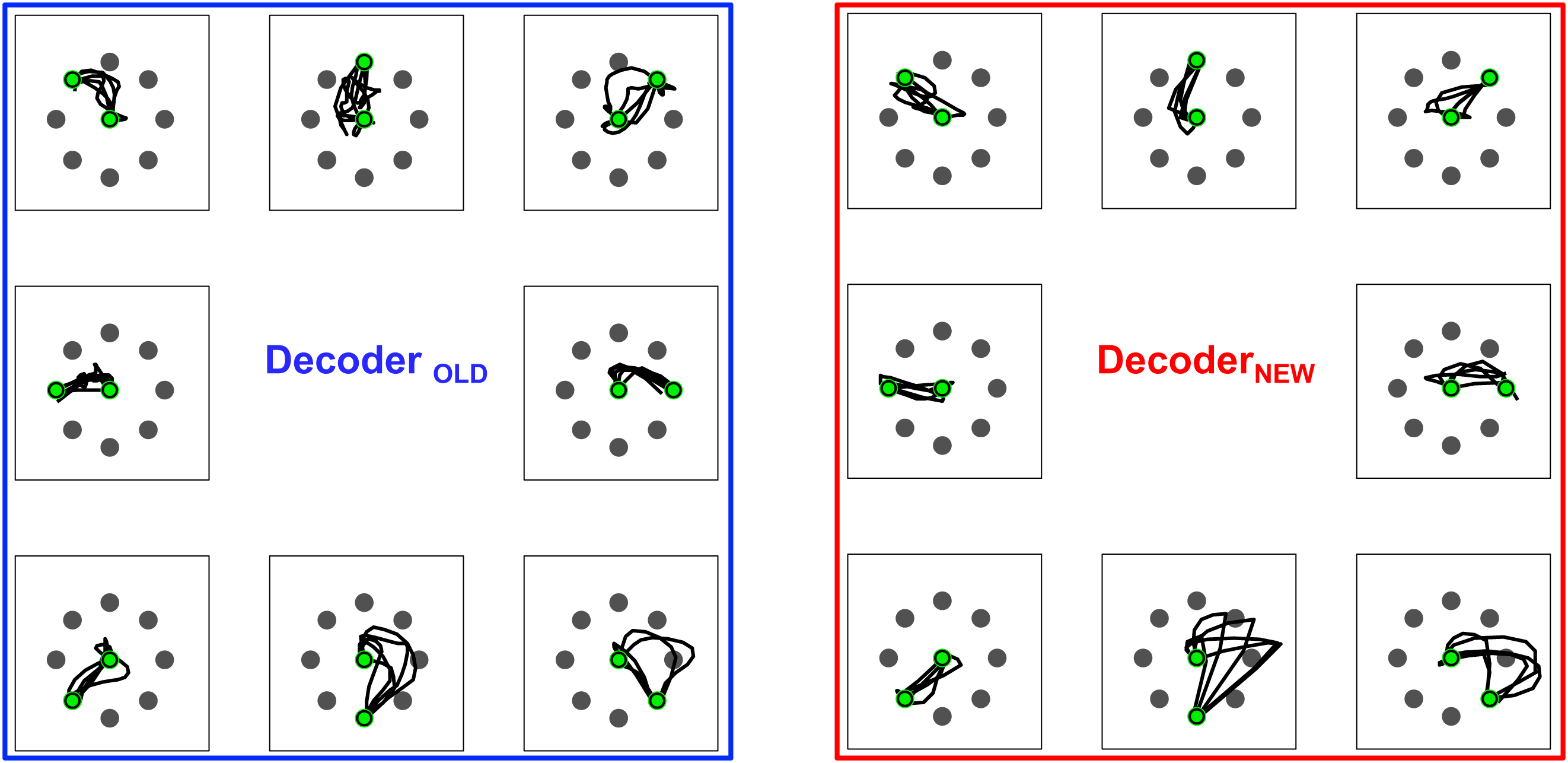

Supplement: Figure S5 — Single-trial cursor trajectories for BC under two decoders. Examples of the single-trial cursor paths during experiments in which two decoders had been simultaneously learned. These represent the neural data analyzed in Figure 8. Each panel shows the cursor path from the center to a target (n = 5). The green circles indicate the active target for a given panel. (0.23 MB TIF) [file pbio.1000153.s005.tif]

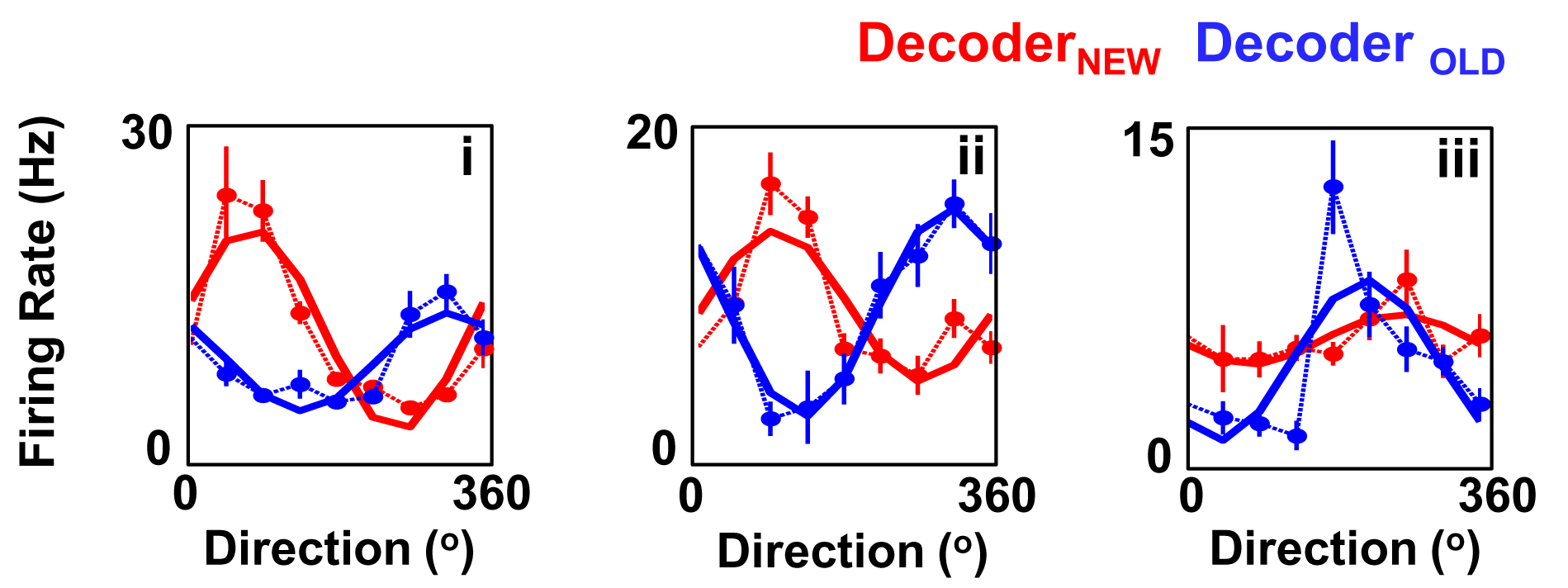

Supplement: Figure S6 — Changes in directional tuning measured with respect to the path of cursor movements. Comparison of the neural tuning functions during BC with two different decoders. The three panels represent a separate analysis of the neural data presented in Figure 8B. Although the tuning functions shown in Figure 8 represent the directional modulation of the neural activity relative to the intended target, the tuning functions shown here represent direction modulation with respect to the actual path of the cursor (first 200 ms). Thus, even after taking into account changes in the path of the cursor, there were changes in the tuning functions. The color scheme is identical to that used in Figure 8. (0.13 MB TIF) [file pbio.1000153.s006.tif]

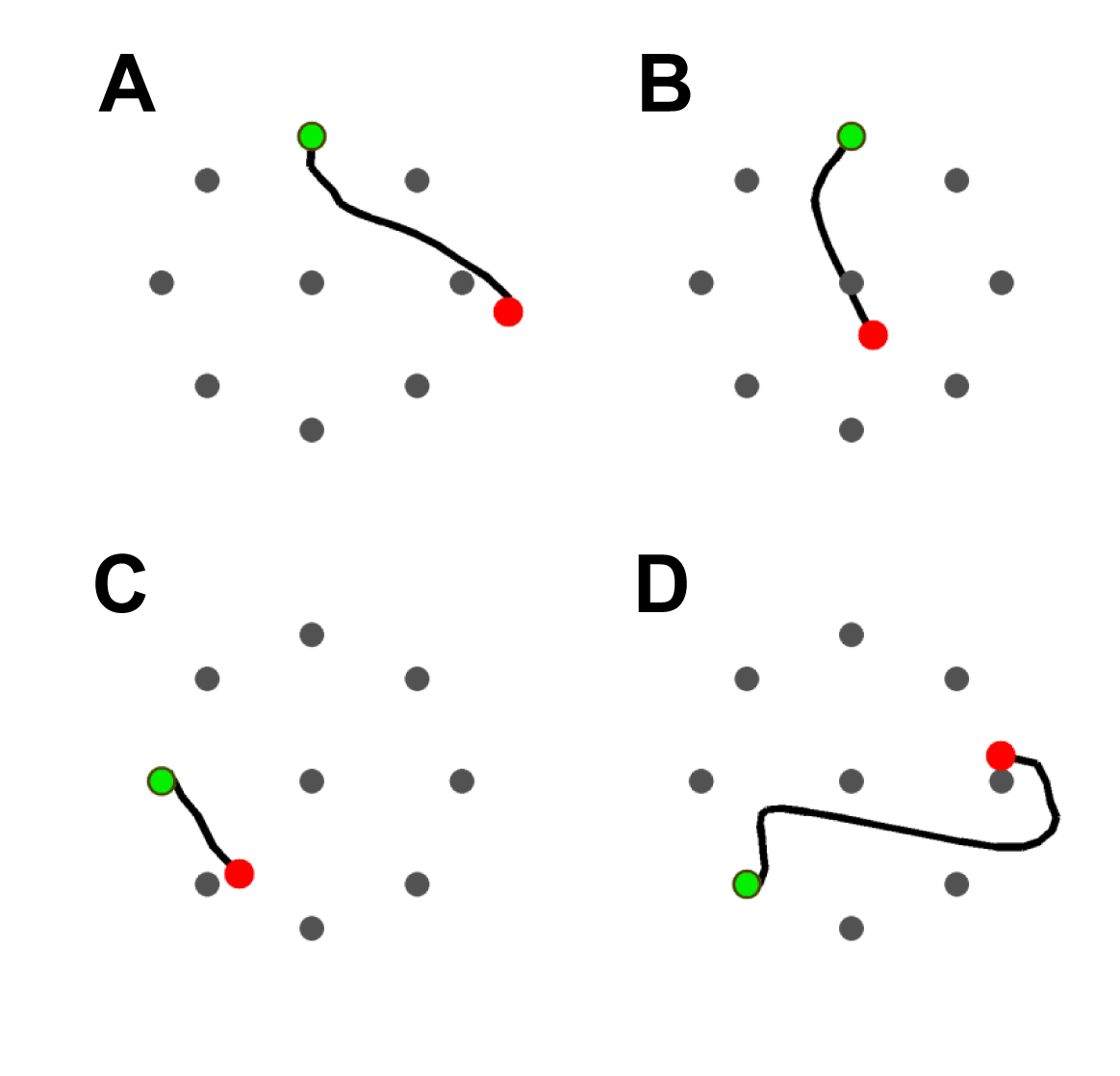

Supplement: Figure S7 — Generalization of prosthetic control beyond the center-out task. Sample trials demonstrating generalization of prosthetic control beyond the stereotyped center-out task. The figure shows how Monkey P can control the cursor from an arbitrary start target. The original task was altered such that the “Start” target (shown in red) randomly appeared within the workspace in an unpredictable pseudorandom order. Although the “End” target (green) remained as prior, novel unrehearsed trajectories were required to reach each of the eight targets. The accuracy level for this new task was typically >85% and did not appear to require extensive relearning. (0.09 MB TIF) [file pbio.1000153.s007.tif]
